# A system dynamics approach to understand Dutch adolescents’ sleep health using a causal loop diagram

**DOI:** 10.1186/s12966-024-01571-0

**Published:** 2024-03-22

**Authors:** Danique M. Heemskerk, Vincent Busch, Jessica T. Piotrowski, Wilma E. Waterlander, Carry M. Renders, Maartje M. van Stralen

**Affiliations:** 1 Department of Health Sciences, Faculty of Science and Amsterdam Public Health Research Institute, Vrije Universiteit Amsterdam, Amsterdam, The Netherlands; 2 Department of Healthy Living, Public Health Service (GGD), Sarphati Amsterdam, City of Amsterdam, The Netherlands; 3 Amsterdam School of Communication Research ASCoR, University of Amsterdam, Amsterdam, The Netherlands; 4 Department of Public and Occupational Health, Amsterdam Public Health Research Institute, Amsterdam UMC Location University of Amsterdam, Amsterdam, The Netherlands

**Keywords:** Adolescent, Action scales model, Causal loop diagram, Complex systems, Sleep, System dynamics, System mapping, Teens, Youth

## Abstract

**Background:**

Healthy sleep is crucial for the physical and mental wellbeing of adolescents. However, many adolescents suffer from poor sleep health. Little is known about how to effectively improve adolescent sleep health as it is shaped by a complex adaptive system of many interacting factors. This study aims to provide insights into the system dynamics underlying adolescent sleep health and to identify impactful leverage points for sleep health promotion interventions.

**Methods:**

Three rounds of single-actor workshops, applying *Group Model Building* techniques, were held with adolescents (*n* = 23, 12–15 years), parents (*n* = 14) and relevant professionals (*n* = 26). The workshops resulted in a multi-actor Causal Loop Diagram (CLD) visualizing the system dynamics underlying adolescent sleep health. This CLD was supplemented with evidence from the literature. Subsystems, feedback loops and underlying causal mechanisms were identified to understand overarching system dynamics. Potential leverage points for action were identified applying the Action Scales Model (ASM).

**Results:**

The resulting CLD comprised six subsystems around the following themes: (1) School environment; (2) Mental wellbeing; (3) Digital environment; (4) Family & Home environment; (5) Health behaviors & Leisure activities; (6) Personal system. Within and between these subsystems, 16 reinforcing and 7 balancing feedback loops were identified. Approximately 60 potential leverage points on different levels of the system were identified as well.

**Conclusions:**

The multi-actor CLD and identified system dynamics illustrate the complexity of adolescent sleep health and supports the need for developing a coherent package of activities targeting different leverage points at all system levels to induce system change.

**Supplementary Information:**

The online version contains supplementary material available at 10.1186/s12966-024-01571-0.

## Background

Healthy sleep is crucial for the physical, and mental wellbeing of adolescents [1–3], as it significantly impacts weight development, emotional wellbeing, school performance, and neurocognitive development [4–6]. Unfortunately, many adolescents do not obtain the recommended 9–11 hours (12–13 years) or 8–10 hours (14–17 years) [7] of good sleep per night [8, 9]. Adolescents in the Netherlands are no exception to this; more than half of Dutch 14–17-year-olds sleep less than recommended minimum of 8 hours a night [10]. Moreover, 24% of 12–16 year-old Dutch adolescents rate their sleep quality as poor and 37% do not wake up rested, with adolescents with pre-vocational secondary education (in Dutch: VMBO^1^) [11] reporting the lowest quality of sleep compared to adolescents with other educational levels [12].

Poor adolescent sleep health is increasingly recognized as a major public health concern and can be considered a complex problem [13]. It emerges via a dynamic interplay of many interconnected causal factors (or: determinants) ranging from biological, economic-, physical-, sociocultural-, and political factors [14, 15]. The complexity of adolescent sleep health is even more pronounced. During adolescence, numerous impactful biological changes occur, including a shift in circadian rhythm due to later release of the ‘sleep hormone’ melatonin, whereby the sleep-wake cycle is shifted leading to a tendency for later bedtimes and waking times. In addition, the homeostatic control of sleep changes during adolescence leading to less ‘sleep pressure’ and staying awake longer [16]. Furthermore, psychosocial changes occur including a growing desire for autonomy (e.g., control over their bedtime routine) and need for peer conformity (e.g., bedtime norms). At the same time, numerous “contextual changes” take place such as transitioning to secondary school, increased educational demands and responsibilities, less parental involvement, increased access and exposure to social media, and other social activities [16, 17]. Together, these biological, psychosocial, and contextual changes impact adolescents’ sleep health in powerful ways.

To date, most preventative interventions designed to address adolescent sleep health have been school-based educational interventions with an exclusive focus on one set of factors (e.g., psychosocial), and not on all factors holistically. Unfortunately, none of these interventions has resulted in significant, lasting effects on adolescent sleep health outcomes [18, 19]. To develop interventions that have structural impact, it is critical to understand adolescent sleep health more holistically, including the interactions between factors, emerging feedback loops, and overarching mechanisms [20, 21]. A ‘systems thinking approach’, which relies on system dynamics methodologies to holistically visualize and interpret complex system dynamics [22] is one such way to understand complex systems, and to detect impactful ‘leverage points’ (i.e., place in the system to intervene) for intervention.

One recent study investigated sleep health from a complex systems perspective and identified feedback loops and mechanisms around the themes: screen use, rules at home and academic pressure [14]. Yet, this paper focused more broadly on obesity-related behaviours (including diet and physical activity) and focused on factors extracted from the literature. To our best knowledge, no studies focused solely on sleep [23] and integrated the perceptions and lived experiences of relevant actors such as adolescents, their parents/caregivers, and professionals. This is an important gap in the literature as the perspectives of these actors may be particularly informative when trying to understand (and potentially change) a complex system such as sleep [24]. Therefore, the aim of the current study is to incorporate these perspectives using system dynamics methods to answer the following research questions:

RQ1: What are the complex system dynamics underlying the sleep health of adolescents with prevocational secondary education?

RQ2: What are leverage points for action to potentially impact adolescent sleep health at different system levels?

## Methods

### Design

Workshops with Group Model Building (GMB) techniques [25–27] were conducted to identify the factors and system dynamics shaping adolescents’ sleep health (RQ1). GMB is a participative system dynamics method whereby actors exchange their perspectives to create a collective understanding of the dynamics of a complex system, and build capacity to think in a systems way [28]. GMB generally results in the construction of a Causal Loop Diagram (CLD), which provides a visual overview of the identified factors impacting a certain complex phenomenon, including their interconnection(s), feedback loops, and larger overarching mechanisms [24, 29].

In addition, CLDs can serve as a starting point for identifying potential leverage points for systems change (RQ2) [30]. We identified those leverage points by using the Action Scales Model (ASM) [31]. The ASM helps to understand a system according to four interconnected ‘levels’ (i.e. events, structures, goals, and beliefs). At the system’s core are beliefs and goals - those entail the system actors’ beliefs and driving forces that in turn determine the system’s purpose. From that, a system’s structure (i.e., the organization of the system causing events to occur) and events (i.e., observable system outcomes) follow to achieve those system goals. To achieve system change, action should be leveraged across all four levels, whereby deeper levels (e.g., goals and beliefs) have more potential to change the system. However, these levels are more difficult to change and require more effort than changing the other levels (e.g., events, structures).

Prior to study onset, ethical approval was obtained by the VU University Medical Ethical Committee (study protocol 2020.0611). 

### Participants and recruitment

Three main actor groups participated in this study, i.e.: adolescents, parents of adolescents, and professionals from education and public health. We focused upon adolescents from pre-vocational secondary education (aged 12–15 years), since this group experiences the poorest sleep health amongst Dutch adolescents [12]. Adolescents were recruited via a youth panel (more than 7500 members between the age of 12 and 24) and social media channels. Parents (or caregivers) were recruited via different social media platforms and with help of professionals from the [removed for blind review]. Educational and public health professionals (i.e., teachers, youth workers, healthy school advisors, youth healthcare professionals, professionals in Public Social Domain) that often interact with adolescents, that have expertise on adolescence, and/or that are knowledgeable on sleep health were recruited to participate via the network of affiliated municipalities and Public Health Services of the project.

Based on existing recommendations on Group Model Building, we aimed to include 10–16 participants per stakeholder group [28, 32]. Ultimately, a total of 23 adolescents (aged 12–15 years, mean age 14 years, 14 girls), 14 parents (aged 36–55 years, 13 female), and 26 professionals with educational and/or public health/social domain background participated in at least one workshop; 15 adolescents and 11 parents participated in more than one session. All participants received information about the workshop prior to participation and provided informed consent. Adolescents provided oral informed consent, and their parents or caregivers provided passive informed consent. In this study we speak of ‘participants’ when we refer to the people that participated in this study. We refer to the term ‘actor’ when we speak about a certain stakeholder group such as adolescents or teachers in general instead of only referring to those adolescents or teachers that participated in the study.

### Nine step-procedure

**Fig. 1:**
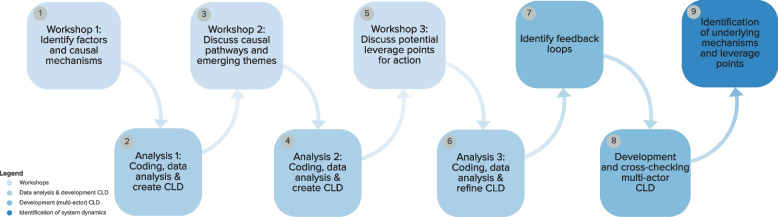
Overview aims per workshop and steps undertaken in creating the multi-actor adolescent sleep health CLD

To build the multi-actor CLD to identify system dynamics, a 9-step procedure was conducted, which consisted of a combination of workshops with GMB techniques, in which data were collected from the three actor groups, the subsequent analysis, development and refining of CLDs, identification of feedback loops and creation of the multi-actor CLD, and analysis of system dynamics (Fig. 1).

#### Workshops with GMB techniques

Three rounds of single-actor workshops were conducted each lasting 90–120 minutes. In the first workshop, factors and causal relationships affecting the different dimensions of sleep health were identified [33], including bedtime (i.e.: the time that one goes to bed); sleep time (i.e., the time that one intends to go to sleep); sleep onset latency (SOL) (i.e., the amount of time it takes to fall asleep); wake up time (i.e., the time one wakes up); and sleep quality (i.e., the subjective judgement of one’s sleep as good or bad) (see Additional file 1 for workshop exercises). In the second workshop, causal pathways were distilled, emergent themes were identified, and conclusions from the prior session were double checked. In the third workshop, actors brainstormed about impactful leverage points for action to change the system, which in turn added a more detailed understanding of certain mechanisms.

Due to the COVID-19 pandemic, all workshops were held online and recorded via the use of the videoconferencing platform Zoom. Given the online setting, workshops were held with a maximum of 7 participants per session. All workshops were facilitated by a team of at least two trained facilitators, one as moderator and another as assistant-facilitator (DH and MvS or VB). Workshops with adolescents were assisted by an external research agency specialized in research with youth including topics as traffic safety and media use [34].

#### Data analysis and development CLD

After each round of workshops, data analysis was conducted and the CLD was developed or refined. For data analysis, recordings of each workshop were transcribed and analysed in MAXQDA Qualitative Data Analysis Software version 2018.2, via an inductive thematic approach using open coding by two independent researchers (DH and MvS or VB). Next, using Kumu software (Kumu relationship Mapping Software 2023), a systems map was created by the research team with all indicated connections between factors and their polarity, meaning one could indicate (1) a positive relationship between variable A and B, or (2) an inverse relationship between A and B.

In the first round of data analysis, factor clusters were identified and organized as emergent subsystems and the initial CLD was created. Based on the data collected in workshop 2 (where causal pathways were discussed in more depth), the second round of data analysis focused on extending and refining the initial CLD. In the third round of data analysis, several refining steps of the CLD were undertaken [14]. Specifically, first, duplicates of factors were deleted and similar concepts (e.g., temperature bedroom, dark bedroom, quiet environment) were merged (e.g,. healthy sleep environment). Second, elements were adapted to fit in a causal pathway wherein we made sure all elements had 1) a neutral labelling (e.g., concentration instead of decreased concentration), 2) were quantifiable (e.g., weekend/vacation was removed as you cannot have ‘more’ or ‘less’ weekend), and 3) changeable (e.g., biological factors such as age, gender and chronotype were removed because this is biologically determined). Third, elements were removed based on a combination of 1) lack of perceived strength of association with other elements and sleep outcomes based on scientific evidence, 2) lack of interconnectedness with other elements and/or 3) when not being part of a feedback loop (once identified). Furthermore, connections were checked for causality to identify whether additional mediating factors or connections were needed. To avoid visualizing too many connections making the CLD unreadable, direct connections were removed if there was also an indirect pathway connecting these elements.

#### Development and cross-checking of multi-actor CLD

After this refinement, it was possible to identify feedback loops with the help of the computer program Vensim (PLE 9.1.1). We identified two types of feedback loops. The first were reinforcing feedback loops (‘R’) indicating that an increase in variable A leads to its further increases. Second were the balancing feedback loops (‘B’), indicating that an increase in a variable ensures its future decrease, thereby creating a balance [21]. At this stage, three separate CLDs were created representing the perspectives of 1) adolescents, 2) parents and 3) professionals.

Following this, the concept multi-actor CLD was created by merging the individual actor CLDs. All variables that were part of a feedback loop or that break or strengthen a certain feedback loop were added to a new ‘integrated multi-actor CLD map’. Where necessary, the CLD was supplemented with missing relationships based on a scientific literature to close certain feedback loops [14].

The factors, connections, feedback loops and subsystems of the multi-actor CLD were checked and discussed by a research consortium consisting of researchers and (public) health care professionals. Prompts included for example: “do you think factors/subsystems/connections are missing?”. After adjustments, the research team reviewed again all new factors and connections to identify possible new feedback loops. In addition, the determined impactful leverage points for action to change the system (workshop 3) were tested among a larger group of young adolescents aged 12–15 via a questionnaire (*N* = 209, *N* = 71 boys, *N* = 132 girls, *N* = 6 neutral) to support the validity and reliability [35].

#### Identification of system dynamics

Lastly, to create an in-depth understanding of the functioning of the system, underlying mechanisms were identified based on the system dynamics within the CLD (i.e., connections, feedback loops and subsystems). Underlying mechanisms can be described as a qualitative description of the overarching process (i.e., mechanism) shaping the system dynamics [14]. Based on the leverage points as identified in the third workshop, the research team used the ASM to determine leverage points that would potentially create structurally lasting impact [31]. Potential leverage points included elements that drive a feedback loop, are part of a feedback loop, are highly connected (e.g., incoming and outcoming connections), or has a strong causal relationship with a sleep dimension [36, 37]. The functioning of each feedback loop within/across subsystem(s) was analyzed for each of the four ASM levels.

## Results

### The CLD

**Fig. 2:**
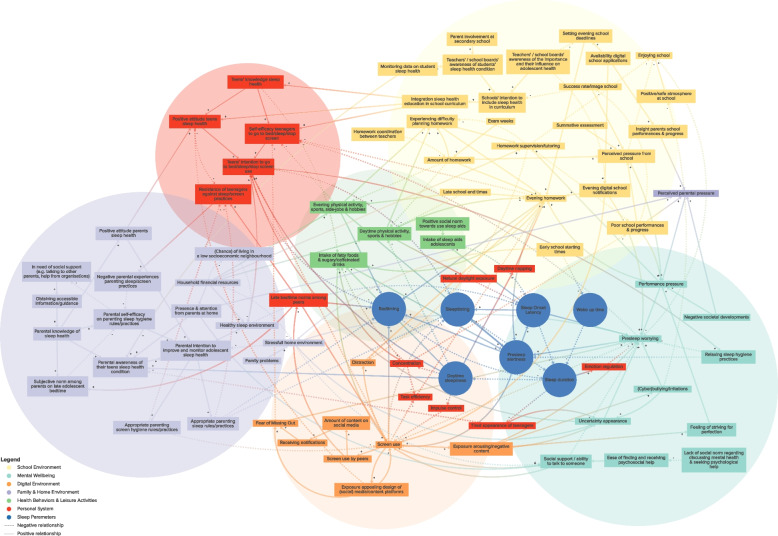
Multi-actor CLD representing system dynamics influencing adolescent sleep health

**Fig. 3:**
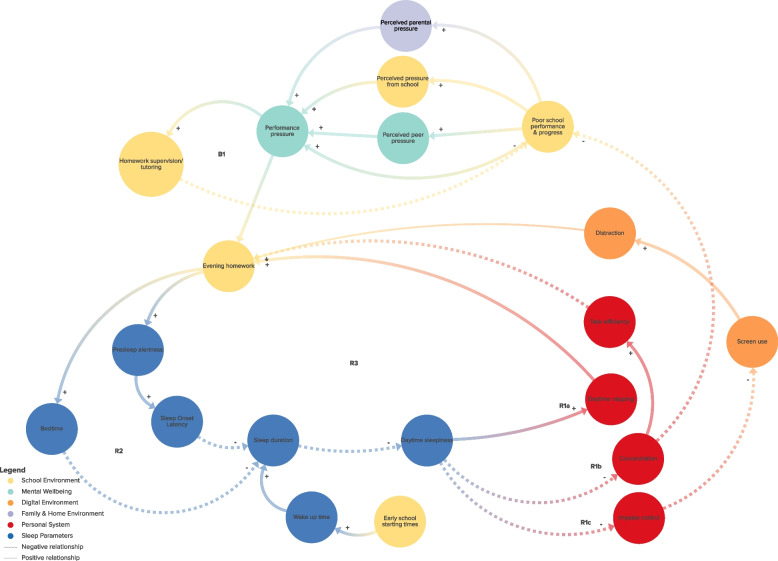
A Multi-actor CLD representing system dynamics related to the school environment (only primary relationships are depicted visually to visualize the identified feedback loops)

Figure 2 illustrates the complete, integrated multi-actor CLD on adolescent sleep health. Six subsystems emerged: (1) the school environment [yellow]; (2) mental wellbeing [mint green]; (3) the digital environment [orange]; (4) the family/home environment [purple]; (5) health behaviors & leisure activities [green]; and (6) personal system [red]. Figure 3A-F illustrate the CLD per different subsystem of which Fig. 3B-F can be found as Additional file 2, 3, 4, 5, 6. In all CLD figures, solid lines represent a positive relationship between two elements (if A increases, B increases, or if A decreases, B decreases), whereas a dotted line represents an inverse, or negative, relationship (if A increases, B decreases or if A decreases, B increases). Table 1 describes all identified system dynamics (i.e., connections, feedback loops and underlying mechanisms) and identified leverage points.

**Table 1:** System dynamics within the CLD: subsystems, feedback loops, mechanisms and leverage points

Subsystem	Feedback loops	Mechanisms	Leverage points
**School environment:** *Evening homework*	**R1:** Evening homework ↑ - presleep alertness ↑ - SOL^*^ ↑ - sleep duration ↓ - daytime sleepiness ↑ - (see a, b, c). **R2:** Evening homework ↑ - (postponing) bedtime ↑ - sleep duration ↓ - daytime sleepiness ↑ - (see a, b, c). *Consequences of daytime sleepiness:* a. Daytime napping ↑ - evening homework ↑ b. Concentration ↓ - task efficiency ↓ - evening homework ↑ c. Impulse control ↓ - screen use ↑ - distraction ↑ /task efficiency ↓ - evening homework ↑	Two overarching mechanisms were identified; (1) adolescents are expected to act more autonomous and demonstrate problem-solving and self-regulatory skills, such as planning their daily activities. However, they are still developing the skills to properly plan and manage their homework as well as leisure activities, causing them to often have to finish their homework in the evening. (2) Dutch regulatory organs judge the quality of a school by how well its students perform on standardized (cognitive) tests. This incentivizes schools to prioritize cognitive learning over e.g., students’ (sleep) health. This results in increased pressure on students to perform, and provide them with more schoolwork.	**Event:** • Reducing evening homework • Aiding students in planning their homework **Structure:** • Aligning school schedules with adolescents’ biorhythm (e.g., starting- and end times, breaks) • Homework coordination between teachers about when and how much homework they give • Prohibiting late-night school deadlines • No digital communication about homework from schools in the evening • Include sleep health as part of the school curriculum • Monitoring data on student sleep health **(current) Goals:** • Providing students with optimal cognitive, educational outcomes (not promoting adolescent (sleep) health) **(current) Beliefs:** • Schools are there to facilitate learning and getting good grades • Lack of teachers’/school board awareness of students sleep health (or mental health) condition • Lack of a belief or awareness that schools have a large responsibility in stimulating sleep health (sleep is something you do at home).
**School environment:** *School performance*	**B1:** Poor school performance ↑ - performance pressure through self-inducing, peers, school and parents ↑ - homework supervision/tutoring ↑ - poor school performance ↓ **R3** Poor school performance ↑ - performance pressure adolescent themselves, peers, from school, parents ↑ - evening homework ↑ - … a. …presleep alertness ↑ - SOL ↑ - … b. …bedtime ↑ -… …sleep duration ↓ - daytime sleepiness ↑ - concentration ↓ - poor school performances & progress ↑		
**School environment:** *School starting times*	No feedback loops identified	School start times were a stand-alone factor in the CLD, yet still of significant importance. Synchronizing them with adolescents’ circadian rhythm benefits their (sleep) health, all-round daytime functioning and consequently school performances.	
**Mental wellbeing:** *Performance pressure*	**R4:** Performance pressure, through self-inducing, peers, school, and/or parents ↑ - presleep worrying ↑ - presleep alertness ↑ - SOL ↑/ wake up time ↓ - sleep duration ↓ - daytime sleepiness ↑ - concentration ↓ - poor school, sports and job performances ↑ - performance pressure ↑ **R5:** Performance pressure ↑ - presleep worrying ↑ - presleep alertness ↑ - screen use ↑ - exposure arousing/negative content ↑ - feeling of striving for perfection ↑ - performance pressure ↑	Today’s adolescent experiences significant peer pressure, continuously reinforced by today’s ever-present social media platforms and influencers, to live ‘the perfect life’, i.e., be ‘successful’, have an active social life, a good reputation and always-perfect, beautiful appearance. Adolescents indicated that as a result they mainly place much pressure on themselves, because they want to comply with this ‘perfect picture’. Pressure from school and parents also appeared to contribute to the feeling of pressure to perform well at school as students are assessed in a summative way and have to choose an educational level at an early stage of their high-school career^**^. Parents themselves feel the pressure to track their child’s progress and achievements via schools’ digital tracking systems/apps, and then ‘stimulate’ them to perform well. The ideology of our current society is also focused on the individual and performance. Parents in turn also perceive this norm and may reflect this on their children.	**Event:** • Providing adolescents with knowledge, awareness, skills and tools to cope with stress & presleep worrying (e.g., relaxing sleep hygiene practices) • Providing more opportunities to talk about mental health (e.g., at school) • No digital communication (e.g., about homework, grades, schedule changes) from schools in the evening • More attention for (cyber)bullying • Providing adolescents with awareness and skills to cope with people portraying a ‘perfect life’ online. **Structure:** • Schools limit their digital communication structures with students and parents • Perceived pressure from parents: restricting parental digital access to school performances • Schools do not focus solely on summative assessment • Requirements/standards for further education are not based on summative assessment only • Mental health is included in the school curriculum **(current) Goals:** • *Adolescents*: complying with the social norm and getting recognition from peers • *Parents*: ensuring the best possible future for their child • *Schools*: maximizing cognitive learning • *Society*: being successful (i.e., dependent one’s education, carrier, income, housing, appearance and social circle) **(current) Beliefs:** • Performance culture (i.e., you are responsible for your own life satisfaction and success) • Norm to not speak about mental wellbeing and seeking professional help
**Mental wellbeing:** *Presleep worrying*	**R6**: Presleep worrying ↑ - presleep alertness ↑ - SOL ↑/wake up time ↓ - sleep duration ↓ - emotion regulation ↓ - presleep worrying ↑		
**Mental wellbeing:** *Screen use & social media*	**R7:** Presleep worrying ↑ - presleep alertness ↑ - screen use ↑ - exposure arousing/negative content ↑ - (see a, b, c, d) *Consequences of exposure arousing/negative content:* a. Presleep worrying ↑ b. Cyberbullying ↑ - presleep worrying ↑ c. Uncertainty appearance ↑ - presleep worrying ↑ d. Feeling of striving for perfection ↑ - performance pressure ↑ **R8**: Presleep worrying ↑ - SOL ↑ - presleep worrying ↑ **B2:** Presleep worrying ↑ - presleep alertness ↑ - screen use ↑ - social support/emotion suppressing ↑ - presleep worrying ↓	Adolescents often use their phone late in the evening and in bed as a means to calm down and ‘switch off’ from all that goes on in their day and the often stressful thoughts that come with it causing. It also enables them to talk to friends about their thoughts and problems. However, conversely, such conversations and social media content also reinforce their worries, feelings of FOMO and keep them attached to their phone, often until deep in the night. Once they notice they can’t get enough sleep that night it even increases presleep worrying.	
**Digital environment:** *Postponing bed- and sleep timing*	**R9**: Screen use ↑ - exposure appealing design social media/game platforms ↑ - screen use ↑ (thereby postponing bed timing/sleep timing). **R10:** Screen use ↑ - exposure appealing design social media/gaming platforms ↑ - positive attitude of teens about sleep health ↓ - intention of teens to go to bed/sleep/stop screen use ↓ - screen use ↑ **B3:** Screen use ↑ - exposure appealing design of social media/gaming platforms ↑ - FOMO ↑ - presleep worrying ↑ - presleep alertness ↑ - SOL ↑ - sleep duration ↓ - tired appearance of teenagers ↑ - uncertainty appearance ↑ - intention of teens to go to bed/sleep/stop screen use ↑ - screen use ↓	The prevailing social norm among adolescents that keeps them up at night is that they are expected to be available 24/7, and to always be online. These norms reinforce screen use but also create late bedtime norms among peers. For adolescents is screen use and social media of extra importance as it is a way for adolescents to explore their identity and express themselves. It is thereby an extension of their personal identity. Social media platforms tap into this need of adolescents to express themselves, explore their identity and search for peer confirmation. By using digital data they create a personalized, cultivated space which is appealing. Their goals is to maximize profits (e.g., via advertisements) and keep you online constantly using captivating mechanisms (e.g., notifications, social media challenges).	**Event:** • Providing adolescents with knowledge, awareness, positive attitude, self-efficacy and skills (e.g., to cope with FOMO and cultivating mechanisms) **Structure:** • *Adolescents*: Screen use (e.g., social media and gaming) are important communication ‘structures’ for teenagers. But also a way to explore their identity and express themselves • *School*: Screen use at school for schoolwork contributes to efficiency • *(social) media companies*: the compulsive ‘structures’ or environments of media platforms by using personal digital data **(current) Goals:** • *Adolescents*: conform with their peers and adhere to the social norm • (*social) media companies*: • Maximize profits by increasing social media and screen use. • Improve and maximize efficiency and convenience of daily life activities as the use of technology can simplify time-consuming tasks **(current) Beliefs:** • Adolescent screen use norm, i.e., it is normalized to be online in the evening, it is expected that you are 24/7 available and that social media is an extension of our personal identity (i.e., many reactions, followers and likes is comparable to popularity and being liked) • Technological innovations are necessary to function in daily life (e.g., school work, entertainment, communication, payments, setting alarm clocks)
**Digital environment:** *Fear of Missing Out & peers*	**R11**: Screen use ↑ - exposure appealing design social media/game platforms ↑ - FOMO ↑ - presleep worrying ↑ - presleep alertness ↑ - screen use ↑ **R12**: Screen use by peers ↑ - amount of content on social media/gaming platforms ↑ - receiving notifications ↑ - FOMO ↑ - presleep worrying ↑ - presleep alertness ↑ - screen use ↑ - screen use by peers ↑		
**Digital environment:** *Peer norms*	**R13**: Screen use by peers ↑ - late bedtime norms peers ↑ - screen use ↑ - screen use by peers		
**Family & Home environment:** *Parenting practices*	**R14**: Appropriate parenting sleep/screen hygiene rules/practices ↓ - resistance of teenagers ↑ - negative parental experiences parenting sleep/screen practices ↑ - parental self-efficacy ↓ - parental intention to improve and monitor adolescent sleep health ↓ - appropriate parenting sleep/screen hygiene rules/practices ↓ (later bedtiming/sleep timing/more screen use) **B4**: Parental self- efficacy ↓ - need of social support (e.g., talking to other parents, help from organisations) ↑ - obtaining accessible information/guidance ↑ - parental knowledge of sleep health ↑ - subjective norm among parents on late adolescent bedtime ↓ - parental self-efficacy ↑	Parents often struggled with their changing role towards their adolescent. Although specific situations may vary per type of parenting style, most parents struggle with their role from manager to coach to mentor through adolescence, especially in context of their child’s sleep health.	**Event:** • Aiding parents with setting, monitoring and enforce appropriate parenting practices (e.g., bedtime, sleep time and screen use) • Aiding parents to be a good role model (e.g., parental evening screen use, bedtime) • Providing parents with knowledge, positive attitude, awareness, self-efficacy and skills to improve sleep health of their children **Structure:** • Busy day to day lives (less priority for parenting practices and less insight into adolescent behavior) • Creating accessible ways for parents to obtain information, guidance and exchanging experiences with other parents • Improving municipal access to financial resources for low socio-economic status families to enable them to create a healthy sleep environment **(current) Goals:** • *Parents***:** their teenagers should grow up happily and in good physical and mental state. • *Parents*: do good as a parent and facilitate the best possible future for their child. • *Parents***:** have a good relationship with their child • *Parents:* having the highest educational level possible for their child **(current) Beliefs:** • Parental norm around sleep and screen parenting • Parental belief that higher educational qualifications of their child enables financial independence and betters their chances in life. • Seeking parental help is perceived as failing as a parent. • Adolescents at this age range should take their own responsibility *or* • Adolescents at this age range can’t take all responsibilities and still need a guiding role from their parents
**Family & Home environment:** *Household factors*	No feedback loops identified	Certain household characteristics significantly influence adolescent sleep health directly (e.g., unsafe neighborhood, housing quality) and indirectly (e.g., financial stress decreasing the priority for parents to set rules).	
**Health behaviors & Leisure activities:** *Sports, hobbies & jobs* *Food, drink and medicine consumption*	**R15**: Daytime physical activity, sports, side-jobs & hobbies ↑ - natural daylight exposure ↑ - SOL ↓ - sleep duration ↓ - daytime sleepiness ↑ - daytime sports, hobbies & jobs ↑ **B5:** Daytime physical activity, sports, side-jobs & hobbies ↑ - evening homework ↑ - presleep alertness ↑ & postponing bedtime ↑ - sleep duration ↓ - daytime sleepiness ↑ - daytime sports ↓ **R16**: Intake of fatty foods & sugary/caffeinated drinks ↑ - presleep alertness ↑ - SOL ↑ - sleep duration ↓ - daytime sleepiness ↑ - intake of fatty foods & sugary/caffeinated drinks ↑ **B6**: Concentration ↓ - intake of fatty foods & sugary/caffeinated drinks ↑ - concentration ↑ **B7**: SOL ↑ - intake of sleep aids adolescents ↑ - SOL ↓	When adolescents have an inadequate sleep health they behave more unhealthy: they eat more fast fatty and sugary foods and they move less during the day because they feel tired. In addition, hormone regulation of ghrelin and leptin become unstable when sleep deprived. Ghrelin levels rises and leptin levels decreases which stimulates eating.	**Event:** • Decreasing adolescents’ consumption of fatty foods & sugary/caffeinated drinks in the evening • Stimulating daytime physical activity • Discouraging evening sports, hobbies & side-jobs • Aiding adolescents with planning homework, side-jobs, sports, hobbies and other leisure activities **Structure:** • Evening working hours of side-jobs and sports activities **Goals:** **-** **(current) Beliefs:** • Norm intake sleep aids
**Personal system** *Psychosocial factors*	No feedback loops identified	Certain personal factors (e.g., knowledge, attitudes, self-efficacy, skills) are important preconditions for proper sleep health. As with many other health-related behaviors, these factors are often insufficient, yet necessary elements for behavior change. Having the intention to perform healthy sleep-related behavior is not enough as adolescents perceive many barriers (i.e., competitive behaviors such as evening homework, sports/hobbies, screen use). One of the effects of these competitive behaviors is that adolescents often think sleep is a waste of their time.	**Event:** • Providing adolescents with knowledge, awareness, positive attitude, self-efficacy and skills to improve their sleep health **Structure:** **-** **Goals:** **-** **(current) Beliefs:** • *Adolescents:* norm about sleep • Sleep is a waste of my time • Sleep is not important • Going to bed late is cool • Peers also go to bed late

^*^SOL Sleep Onset Latency

^**^ Students at pre-vocational secondary education choose a specialisation at the latest at the end of the 2nd school year

## Complex system dynamics (RQ1)

### Feedback loops

RQ1 asks what are the complex system dynamics underlying the sleep health of adolescents? In total, we identified 23 feedback loops: 16 reinforcing and 7 balancing loops (Table 1). For example, feedback loops R1 and R2 illustrate how evening homework impacts adolescent sleep health (Fig. 3A). Doing homework late at night stimulates mental arousal and alertness, making it harder to fall asleep soon after. Parallel to this, it takes up time, postponing bed- and sleep time, resulting in shorter sleep duration and more daytime sleepiness. In different ways (e.g., daytime naps, decreased concentration, and being more easily distracted through lessened impulse control), such daytime sleepiness decreases focus on school work. All this results in now having to do even more homework than before to keep up in school, thus completing a vicious cycle.

Rather than feedback loops within a certain subsystem, numerous feedback loops connecting subsystems were identified (Fig. 2). One such a cross-subsystem feedback loop for example went as follows: schools often send out communications late in the evening (e.g., on grades, homework, or schedule changes), which are usually immediately received by teens and their parents via push notifications from school applications. In this way, receiving a bad grade causes immediate arousal and/or discussions with parents and/or peers, thus causing much pre-sleep worrying. Following this, as a coping strategy, teenagers check their phone, further shortening their night and leaving them more tired the next day. Such daytime sleepiness further worsens their school performances, strengthening this cycle (R7/R8). Such a cross-subsystem feedback loop illustrates the relationship between the school environment, digital environment, the family/home environment and the personal system.

When developing the CLD to identify system dynamics, including the perceptions of multiple actors provided detailed insight into specific mechanisms. Teachers and healthy school advisors provided detailed understanding of mechanisms within the school environment, professionals on driving system forces on macro level (e.g., social media industry), and parents on the influence of parenting practices, the home environment, but also the living environment of adolescents. For example, parents indicated the existence of digital evening school notifications while adolescents did not mention this at first. In addition, the different perspectives enabled to gain insight into current norms, beliefs, experiences, and perceptions. For example, some adolescents stated that blue light gave them the feeling of tired eyes causing them to fall asleep faster. Also, some adolescents indicated that screen use and receiving notifications can decrease their feelings of missing out and gives them rest because they are up to date about what is happening online. Lastly, some adolescents indicated that evening intake of sugary and caffeinated drinks and food has no effect on their presleep alertness.

### Underlying mechanisms

We found several mechanisms per subsystem explaining why these subsystems, feedback loops and connections exist (Table 1). Take for example, the school system. Here, we identified the mechanism that schools aim to maximize cognitive learning, because they must meet the requirements (educational core objectives) that determine what students should know and be able to do. Schools are assessed for compliance with these requirements and the cognitive achievements of students by regulating organizations. This underlying mechanism causes the increased pressure to perform at school, more schoolwork and thus more evening homework activities, and less prioritization for health-related topics at school. This underlying mechanism also elucidates why, for instance, later school starting times are not exclusively influenced by schools’ awareness of sleep health. Rather, this factor is influenced by a multitude of determinants and causal mechanisms determining the adoption and implementation of later school starting times which is beyond the scope of adolescent sleep health and thus the current study.

## Leverage points for system change (RQ2)

RQ2 asks what are the leverage points for structural system changes to potentially impact adolescent sleep health? In total, we identified 60 leverage points for systems change across the ASM levels within several contexts related to the identified subsystems, such as e.g., the school environment targeting (e.g., delayed school starting times); digital environment (e.g., changing 24/7 screen use norms); and the societal context (e.g., changing current goals and beliefs around performance culture). We identified and summarized all leverage points in Table 1. Herein, we distinguished between (visible) events (*N* = 16) and structures (*N* = 18) versus underlying system goals (*N* = 12) and beliefs (*N* = 14). Leverage points at event and structure level were often visible within the CLD and were derived from the results of the third workshop where potential leverage points for action were discussed. Insight into leverage points at goal and belief level were provided by information from the actor groups about prevailing norms and beliefs, and the identification of underlying mechanisms by the research team. Most of the leverage points across the levels are interconnected and reinforce their potential impact of changing the system. For example, to improve sleep duration different efforts at all levels are required to maximize their impact. On the level of event it is important to create awareness and improve knowledge among adolescents about appropriate sleep duration. However, also school starting times should be more aligned with the biorhythm of adolescents to facilitate good daytime functioning and school performances, which requires a change in the organization of a school day (structure). To attain this, actions on goal and belief level are preferred: schools need to view (sleep) health as being as vital as focusing on cognitive outcomes directly (goal) and they should believe that they can play an important role in the sleep health status of adolescents (belief).

## Discussion

This study aimed to provide insights into the system dynamics underlying the sleep health of adolescents with prevocational secondary education (RQ1), and to identify potential leverage points for change at all levels within this system (RQ2). A Causal Loop Diagram was created and six subsystems were identified – namely, the school environment, mental wellbeing, the digital environment, the family/home environment, healthy behaviors and leisure activities, and the personal attributes – which together shape the sleep health system of adolescents. Across these six subsystems, we identified: a) 23 different feedback loops (i.e., 16 reinforcing and 7 balancing) – demonstrating the interconnectedness of the systems, b) underlying mechanisms of these feedback loops, and c) 60 potential interconnected leverage points for systems change across different system levels (i.e., events, structures, goals and beliefs).

The identified factors or subsystems in our CLD align with existing evidence from empirical studies [14, 38–41]. For example, the ‘Perfect Storm Model’ highlights that insufficient adolescent sleep health is a result of bioregulatory and psychosocial factors, such as changes to the homeostatic system, school starting times, socioeconomic status, digital media use, social engagements and caffeine intake [16]. Additionally, Grandner et al.’s (2019) ‘Social-Ecological Model of Sleep Health’ explains how individual, social, and societal level determinants influence sleep health, highlighting that individual level-factors (e.g., individual genetics, beliefs and attitudes about sleep, sleep-related behaviors) are interconnected with broader social- (e.g., home, family, work, school, neighborhood, religion, culture, socioeconomic status) and societal factors (i.e., globalization, 24/7 society, geography, public policy, technology and economics). With both models, we find our own results which highlight the complex interconnectedness that underlies sleep health. At the same time our study adds an in-depth understanding of the interplay between factors and subsystems, and of the underlying emerging system dynamics that shape (the functioning of) the system. While biological factors (e.g. gender, age, chronotype, hormonal influences) are relevant, they were not included in this CLD because they did not meet our system boundary criteria, which focus on including modifiable factors to eventually identify potential impactful leverage points for systems change. Nevertheless, biological factors might be taken into consideration to attain a comprehensive understanding of the adolescent sleep health system. Furthermore, we confirmed and extended results found within the literature based CLD developed by Waterlander et al. (2020). For example, we also identified a reinforcing feedback loop around ‘rules at home’ as this can cause resistance of teenagers and decrease the self-efficacy of parents to make appropriate parenting bedtime/sleep/screen hygiene rules with their teenager.

Our study provides a new contribution to today’s literature by adding the perspectives of relevant actors and emphasizes the need for a coherent systems approach with intervening efforts addressing *several* system dynamics within different settings to initiate systems change and to promote adolescent sleep health. Thus far, most preventative intervention efforts are often focused on single elements such as personal determinants (e.g., school education or relaxation techniques), parenting styles and practices or delaying school starting times [19]. While this is an important first step, our findings points towards extending these levels to also addressing deeper levels, such as the beliefs of society including adolescents, parents, teachers/school boards, policy makers and politicians. In that regard, we recommend the ASM model as a useful tool to understand deeper system levels and to identify leverage points for system change on all levels.

## Looking ahead

### Empirically

This study serves as an exemplar for applying system dynamics methodologies to understand and address complex public health problems. We found it valuable to gain insights into the perspectives of our actor groups (i.e., adolescents, parents, professionals) on this complex problem as it deepened our understanding of specific causal mechanisms at play. Overall, there were many similarities between the perspectives of our different actor groups on contributors and mechanisms of adolescent sleep health, whereby each actor group could provide detailed insights into specific subsystems, certain causal mechanisms and impactful leverage points. Some adolescent perceptions (e.g., the positive impact of blue light, receiving notifications and caffeine intake on falling asleep) appeared to contradict the existing, primarily quantitative, scientific evidence (e.g., the negative impact of blue light, receiving notifications and caffeine intake on falling asleep). This highlights the added value of including perspectives when trying to understand health-related topics and developing interventions. We recommend that future research also strive to involve multiple actors in the process of creating a systems maps (CLD) and use this information in combination of existing evidence to yield the most robust picture of the system and to identify leverage points that are impactful and supported by the actors involved.

The COVID-19 pandemic forced us to conduct the workshops online. To address potential challenges associated with online workshops, we maintained smaller group sizes (approximately 7 people per session). This still aligns with the recommended range of at least 5 participants in GMB workshops for optimal group dynamics [28]. While a few participants, mostly parents, needed additional support in using online collaborations tools like ‘Miro Board’ or ‘Mentimeter’, the majority expressed enthusiasm about participating and expressed interest in being included in potential future follow-ups. An advantage for participants was that they did not had to travel, thereby reducing the barrier for participation.

Furthermore, we found the combination of the Action Scales Model with CLD in our study useful to better understand the deeper levels of the system and to identify leverage points for system change on all levels in a structural manner. Without the ASM approach, leverage points on ‘goal’ and ‘belief’ level would otherwise be neglected as these are not always represented in a CLD [31]. To that end, we encourage future studies to consider the use of the ASM framework to both better understand the working of the system and to identify leverage points for change at all levels.

Related to the generalizability of our findings, we aimed to understand the system dynamics shaping adolescent sleep health within the Dutch context. We expect that the dynamics found seem to be relevant to other contexts as well, although the influence of contextual factors may lead to the identification and prioritization of other leverage points in other settings such as other countries, target populations and specific subgroups. For example, we acknowledge that certain mechanisms (i.e., factors, connections and identified feedback loops) are similar but could be more prominent within specific adolescent subgroups based on age, gender, educational level and culture. When aiming to understand the specific system dynamics at play within another country, target population or specific subgroup, repeating the process is probably required. We hope our CLD can be a starting point for that.

Lastly, because a CLD is a static representation of a current system in a Dutch context, it should be recognized that systems are constantly evolving and adapting when changes occur [21]. In that way, we recommend that – as the system changes – that procedures are followed to create a revised CLD to determine if and how leverage points may have been impacted. In this way, interventions can remain sensitive to the dynamic needs of the system.

### In practice

While there is much to be said about the value of this process empirically, it is also reasonable to ask what are the practical implications now from this work. Our system dynamics approach revealed several implications for practice, including insights into possible leverage points for action and belonging unintended and unexpected consequences of future actions. For example, delaying school starting times has a directly positive effect on sleep duration [42]. However when introducing this measure, it is important to also consider the ending times of school, as delayed school ending times can reinforce evening homework and potentially reduces the positive effects of delayed starting times on sleep duration (i.e., an unintended consequence). Therefore, when delaying school starting times, other actions such as homework planning and support during school hours should be implemented simultaneously to prevent these unintended consequences.

To be sure, like other system dynamics studies [14], the dynamics identified here have not yet been empirically tested. Our findings, in general, are consistent with existing empirical insights in the field, but nonetheless, we recommend that the findings be interpreted with a reasonably critical lens until effects-based scholarship can augment these findings. We encourage researchers and practitioners to build on this work, using these findings to shape actions targeting the identified dynamics and investigating the extent to which these patterns hold in practice.

The leverage points identified in this study can inform the development of a ‘whole systems approach’, which is needed to structural implement proper policies, actions and interventions targeting the leverage points. Currently, there is no whole systems approach addressing sleep health. However, such useful and successful approaches have been designed and implemented e.g., on obesity prevention [43, 44]. Prior to such implementation efforts, coherent policies, actions and interventions should be co-created with stakeholders by having those 60 leverage points serve as input. While our results confirm there is no ‘silver bullet’, as a starting point, we would recommend leverage points that are e.g., demand-driven (e.g., schools want to do ‘something’ with the topic sleep but lack the tools to do so). With this demand-driven starting point, we recommend making use of existing networks and resources available (e.g., an existing network that have the same vision, time and money to collaborate in a lobby for regulating social media platforms) and aligning with the current policy agenda (e.g., improving mental health of youth). By capitalizing on such a window of opportunity, one is better positioned to affect change.

## Conclusion

Findings of this study contribute to the in-depth understanding of the system dynamics shaping adolescent sleep health. Causal mechanisms and feedback loops were found within and across subsystems, with 6 critical subsystems identified: 1) the school environment: with dynamics around evening homework, school performance, 2) mental wellbeing: with dynamics around performance pressure, presleep worrying, social media, 3) digital environment: with dynamics around screen use, social media content, peer norms, 4) the family and home environment: with dynamics around parenting, household factors, 5) lifestyle behaviors: with dynamics around daytime PA, food/drink/medicine consumption, and 6) the personal system: with dynamics around psychosocial factors. Using the Action Scale Model, we identified 60 leverage points for systems change at four levels (i.e., events, structures, goals, beliefs). We recommend to develop a coherent whole systems approach including interventions targeting multiple system dynamics related to the identified subsystems (e.g., providing adolescents with knowledge, awareness, skills and tools to cope with stress, planning homework & leisure activities, delaying school starting times, no digital evening school communication, providing parental parenting support during adolescence, changing 24/7 screen use norms, changing current goals and beliefs around performance culture). This study highlights that no single solution can bring about significant and lasting system change. Instead, a package of actions and policies that target various leverage points across different levels and settings, both within and beyond the public health domain, are necessary for effective and sustainable system change.

### Supplementary Information

**Additional file 1:.** Description of workshop tasks.

**Additional file 2:** Multi-actor CLD representing system dynamics related to adolescent mental wellbeing (only primary relationships are depicted visually to visualize the identified feedback loops).

**Additional file 3:** Multi-actor CLD representing system dynamics related to the digital environment (only primary relationships are depicted visually to visualize the identified feedback loops).

**Additional file 4:** Multi-actor CLD representing system dynamics related to the family & home environment (only primary relationships are depicted visually to visualize the identified feedback loops).

**Additional file 5:** Multi-actor CLD representing system dynamics related to health behaviour & leisure activities (only primary relationships are depicted visually to visualize the identified feedback loops).

**Additional file 6:** Multi-actor CLD representing system dynamics related to the personal system (only primary relationships are depicted visually to visualize the identified feedback loop)s.

## Data Availability

The datasets used and/or analysed during the current study are available from the corresponding author on reasonable request.

## Abbreviations

ASM: Action scales model
CLD: Causal loop diagram

## Footnotes

1 **The Dutch education system:** Pupils entering secondary education in the Netherlands (usually around 12 years of age) are streamed according to aptitude into one of four forms of schooling: practical education (PrO), pre-vocational education (*vmbo*), senior general secondary education (*havo*), and pre-university education (*vwo*).
