# Supplementary material for: A system dynamics approach to understand Dutch adolescents’ sleep health using a causal loop diagram
Source: Int J Behav Nutr Phys Act. 2024 Mar 22;21:34. doi: 10.1186/s12966-024-01571-0 (PMC10958857; doi:10.1186/s12966-024-01571-0)
Supplement: Supplementary file 1 — Additional file 1:. Description of workshop tasks. [file 12966_2024_1571_MOESM1_ESM.docx]

**Additional file 1: Description of workshop tasks**

| **Workshop*** | **Task name** | **Task description** |
| --- | --- | --- |
| **Workshop One**  Aim: Identifying factors influencing sleep health (i.e. bedtime, sleep time, sleep onset latency, wake up time and sleep quality). | Adolescent sleep health | Explanation of health problem: declining sleep duration and sleep quality of Dutch adolescents. |
|  | Brainstorm | Working individually, participants brainstormed about the focus statement: “why do adolescents sleep good or bad?”. They were encouraged to write down their answers using the online collaboration tools Miro Board^i-ii^ or Mentimeter^iii^. |
|  | Group discussion | Subsequently, the answers were discussed within the group to obtain more in-depth understanding about the factors written down and the relationships between the factors and sleep health and/or between factors, and the type of relationship (i.e. positive or negative). With this, there was also room for discussion, additions and adaptations. |
|  | Timeline ^i^ | Adolescents were asked to fill in a timeline of their day using Miro Board. This served as a starting point for conversation about the influence of their sleep-related behaviour and their living environment on sleep health, and factors influencing their sleep-related behavior. |
|  | Brainstorm bedtime, sleep time, waking up time, sleep quality | Facilitator explained the differences between bedtime, sleep time and waking up time and the concept sleep quality. Thereafter, participants were encouraged to individually brainstorm what factors influences bedtime, sleep time, waking up time and sleep quality of adolescents using Miro Board^i^ or Mentimeter^ii-iii^. |
|  | Introduction to systems thinking | Facilitators explained the systems thinking concept ‘causal connections’. |
|  | Causes and consequences discussion | On the Miro Board ^i-ii^ the facilitator collected all factors of the previous exercise and placed them into one main diagram. In a group discussion, participants brainstormed about the causes and consequences of the factors written down and were encouraged to make links between causes and consequences to form beginnings of feedback loops. During the workshops with parents this exercise was done via conversation techniques: asking questions to identify causes and consequences. |
| **Workshop Two**  Aim: Obtaining more in-depth information about the causal pathways of factors affecting sleep health and sleep-related behavior to form and close causal loops*.* | Presenting and introducing linear map (initial CLD) | Facilitator presented the linear map as result of workshop one showing and explaining causes and consequences, and subsystems. |
|  | Group discussion | Facilitator asked questions about the consequences of inadequate sleep in order to close feedback loops. |
|  | “Traffic lights” technique | Facilitator asked participants to look for a red, yellow and green object. The facilitator asked questions related to factors and/or subsystems of the initial CLD that were unclear in order to: obtain the perceived importance of the factors and subsystems affecting sleep health and double check conclusions from the prior session. Participants answered by holding their object in front of the camera. |
|  | Group discussion | After each ‘traffic light’ question a group discussion took place in order to obtain more in-depth information about the causes and consequences of these factors, to add other factors or connections to the CLD, and correct any errors or misinterpretations from the initial CLD. These discussions also entailed questions based on the system science exercise ‘Graphs over time’: ‘Are there changes over time that may influenced the sleep health of adolescents?’(Gerritsen et al. 2020). |
| **Workshop Three**  Aim: Identify potential impactful leverage points for action in order to improve sleep health of adolescents. | Recap previous workshops: introduction problem and presenting CLD | Facilitator explained the problem and showed the CLD as results of previous workshop(s). |
|  | Identifying impactful leverage points for action to improve sleep health of adolescents | After explaining the CLD participants brainstormed about impactful leverage points for action to change the system. Participants brainstormed individually about the question “If you were the boss of the Netherlands, what would you do to improve the sleep health of adolescents?” They wrote down their answers using the online program Mentimeter. Subsequently, answers were discussed in the group and participants were asked to explain on what factor(s) the action has its influence. |
|  | Priority of actions | Participants were asked to fill in an online poll: “what subsystem do you find most important for improving adolescent sleep health?”. This poll resulted in a ranking of most important subsystems. |
|  | Identifying impactful leverage points for action per subsystem of the CLD | Following the ranking from the previous actions, per subsystem participants brainstormed about impactful leverage points for action of the subsystem in question that would improve adolescent sleep health from their perspective. They were encouraged by the facilitator to think on micro-, meso-, and macro-level. Subsequently, answers were discussed in the group. |

^*^All workshops started with an introduction and icebreaker and ended with a closing.
