# Supplementary figures and images for: A system dynamics approach to understand Dutch adolescents’ sleep health using a causal loop diagram
Source: Int J Behav Nutr Phys Act. 2024 Mar 22;21:34. doi: 10.1186/s12966-024-01571-0 (PMC10958857; doi:10.1186/s12966-024-01571-0)

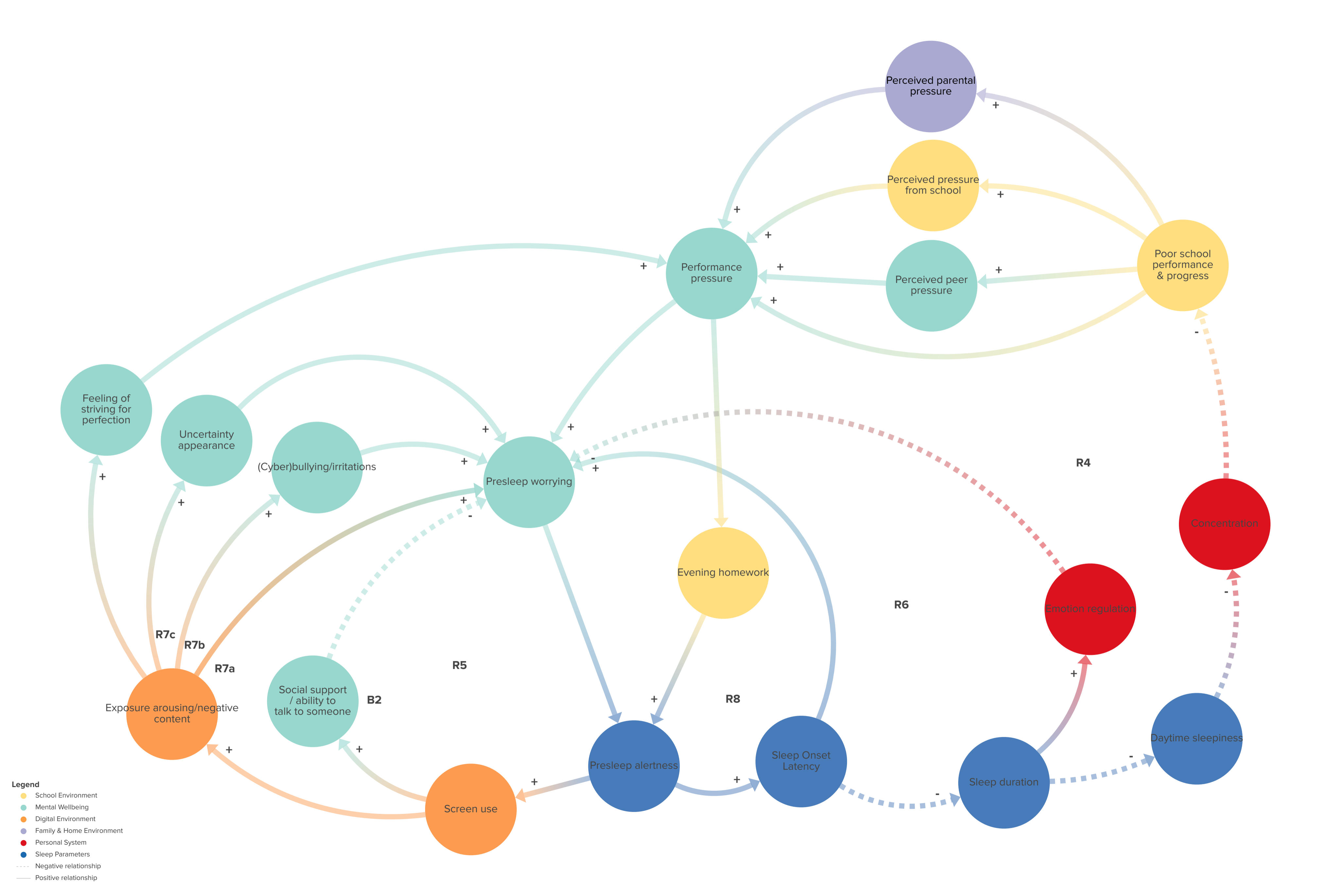

Supplement: Supplementary file 2 — Additional file 2: Multi-actor CLD representing system dynamics related to adolescent mental wellbeing (only primary relationships are depicted visually to visualize the identified feedback loops). [file 12966_2024_1571_MOESM2_ESM.pdf]

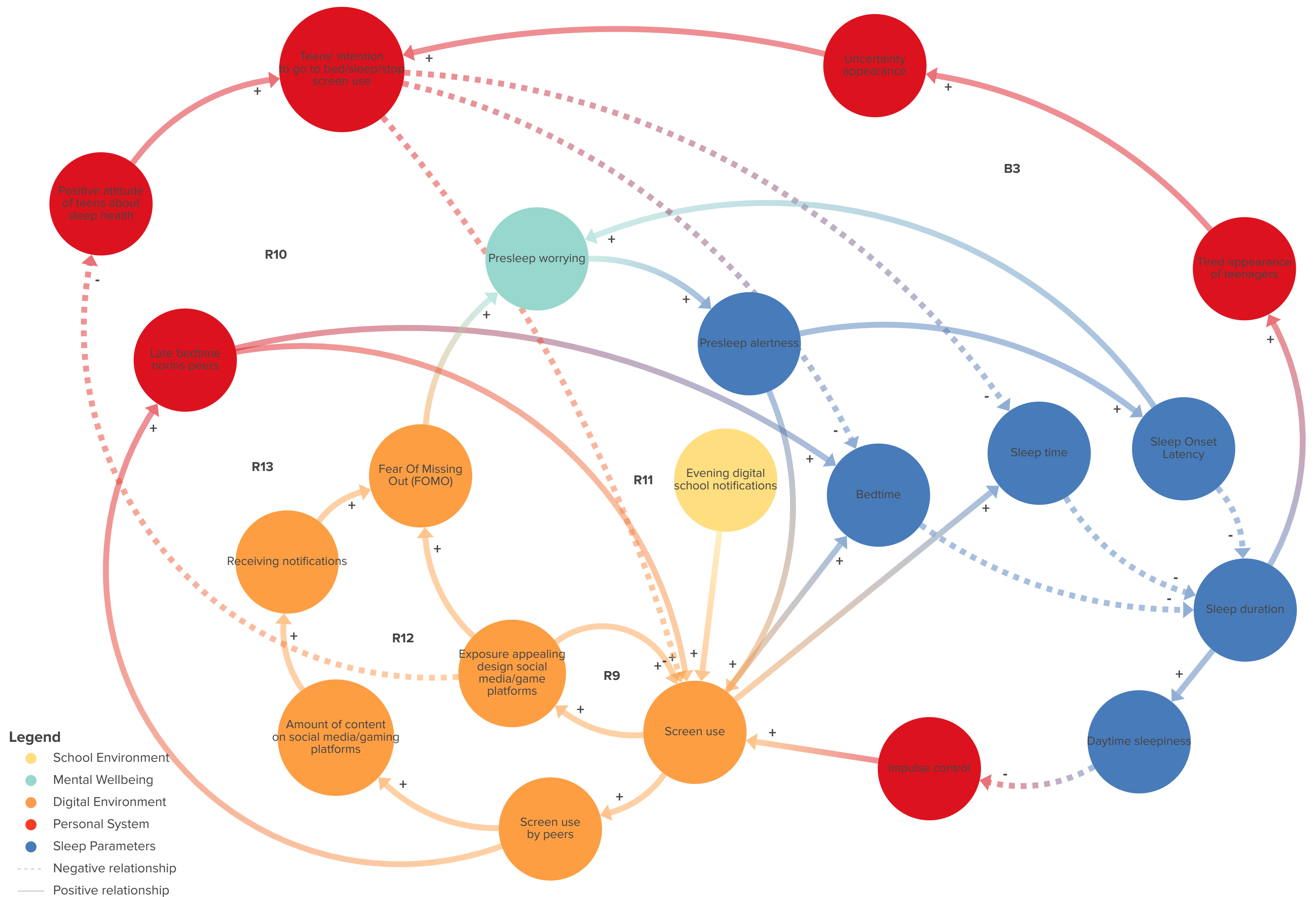

Supplement: Supplementary file 3 — Additional file 3: Multi-actor CLD representing system dynamics related to the digital environment (only primary relationships are depicted visually to visualize the identified feedback loops). [file 12966_2024_1571_MOESM3_ESM.pdf]

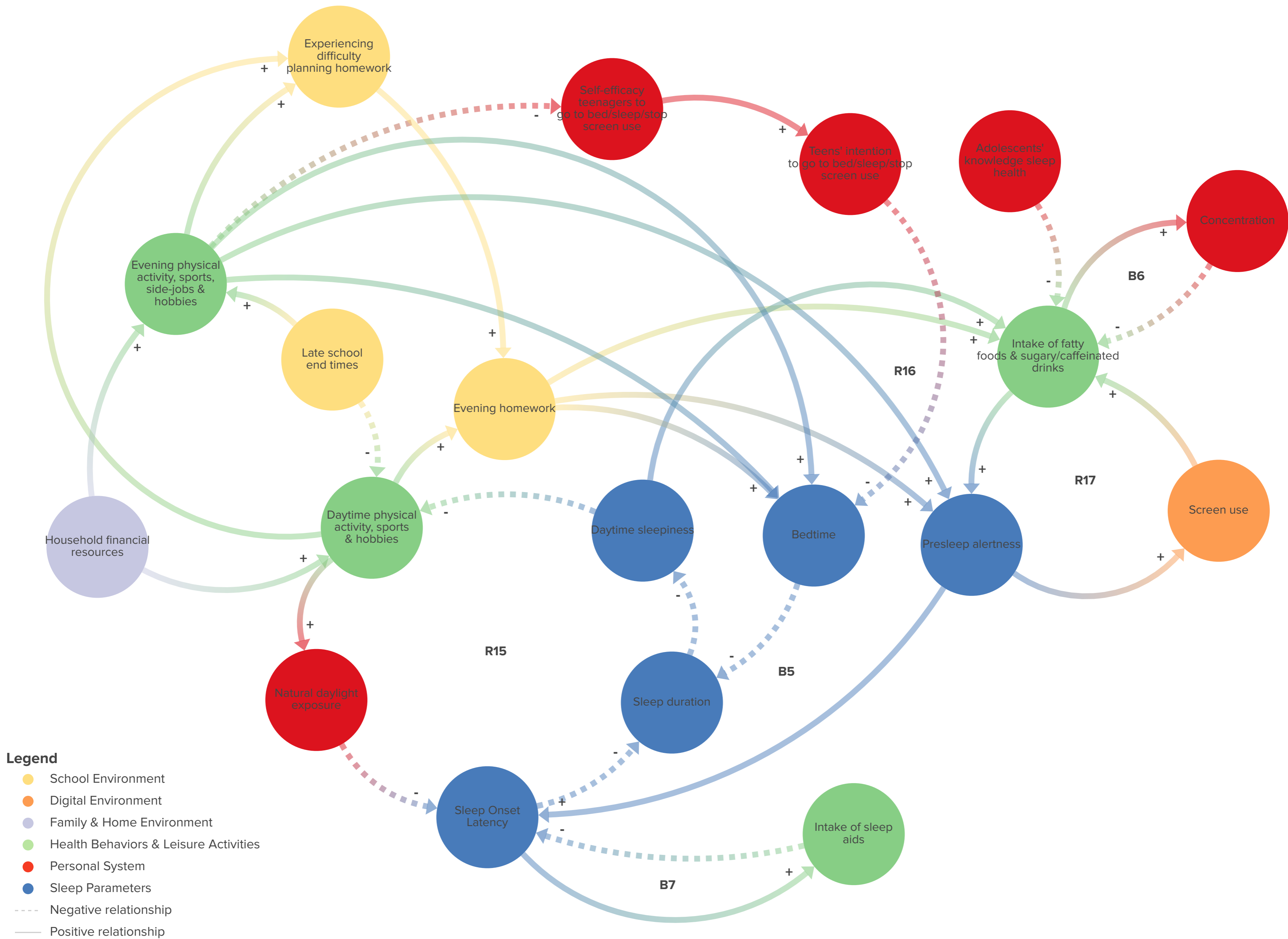

Supplement: Supplementary file 5 — Additional file 5: Multi-actor CLD representing system dynamics related to health behaviour & leisure activities (only primary relationships are depicted visually to visualize the identified feedback loops). [file 12966_2024_1571_MOESM5_ESM.pdf]
