# Supplementary material for: A system dynamics approach to understand Dutch adolescents’ sleep health using a causal loop diagram
Source: Int J Behav Nutr Phys Act. 2024 Mar 22;21:34. doi: 10.1186/s12966-024-01571-0 (PMC10958857; doi:10.1186/s12966-024-01571-0)
Supplement: Supplementary file 4 — Additional file 4: Multi-actor CLD representing system dynamics related to the family & home environment (only primary relationships are depicted visually to visualize the identified feedback loops). [file 12966_2024_1571_MOESM4_ESM.pdf]

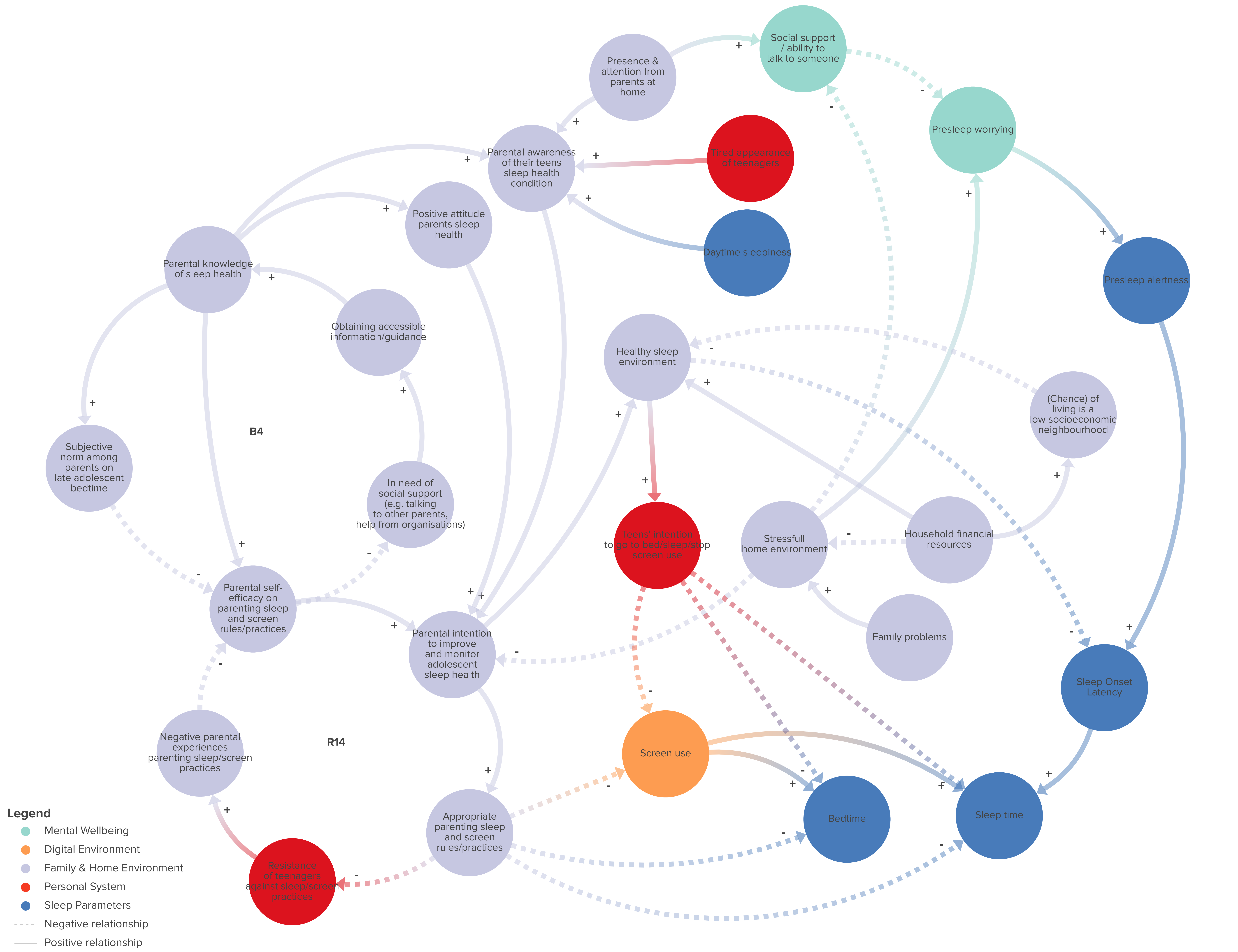

Presence & attention from parents at home
Social support / ability to talk to someone
Presleep worrying
Tired appearance of teenagers
Parental awareness of their teens sleep health condition
Positive attitude parents sleep health
Daytime sleepiness
Presleep alertness
Parental knowledge of sleep health
Obtaining accessible information/guidance
Healthy sleep environment
(Chance) of living is a low socioeconomic neighbourhood
B4
Subjective norm among parents on late adolescent bedtime
In need of social support (e.g. talking to other parents, help from organisations)
Teens' intention to go to bed/sleep/stop screen use
Stressfull home environment
Household financial resources
Parental self-efficacy on parenting sleep and screen rules/practices
Parental intention to improve and monitor adolescent sleep health
Family problems
Sleep Onset Latency
R14
Negative parental experiences parenting sleep/screen practices
Screen use
Bedtime
Sleep time
Appropriate parenting sleep and screen rules/practices
Resistance of teenagers against sleep/screen practices
Legend
Mental Wellbeing
Digital Environment
Family & Home Environment
Personal System
Sleep Parameters
Negative relationship
Positive relationship
