# Supplementary material for: A system dynamics approach to understand Dutch adolescents’ sleep health using a causal loop diagram
Source: Int J Behav Nutr Phys Act. 2024 Mar 22;21:34. doi: 10.1186/s12966-024-01571-0 (PMC10958857; doi:10.1186/s12966-024-01571-0)
Supplement: Supplementary file 6 — Additional file 6: Multi-actor CLD representing system dynamics related to the personal system (only primary relationships are depicted visually to visualize the identified feedback loop)s. [file 12966_2024_1571_MOESM6_ESM.pdf]

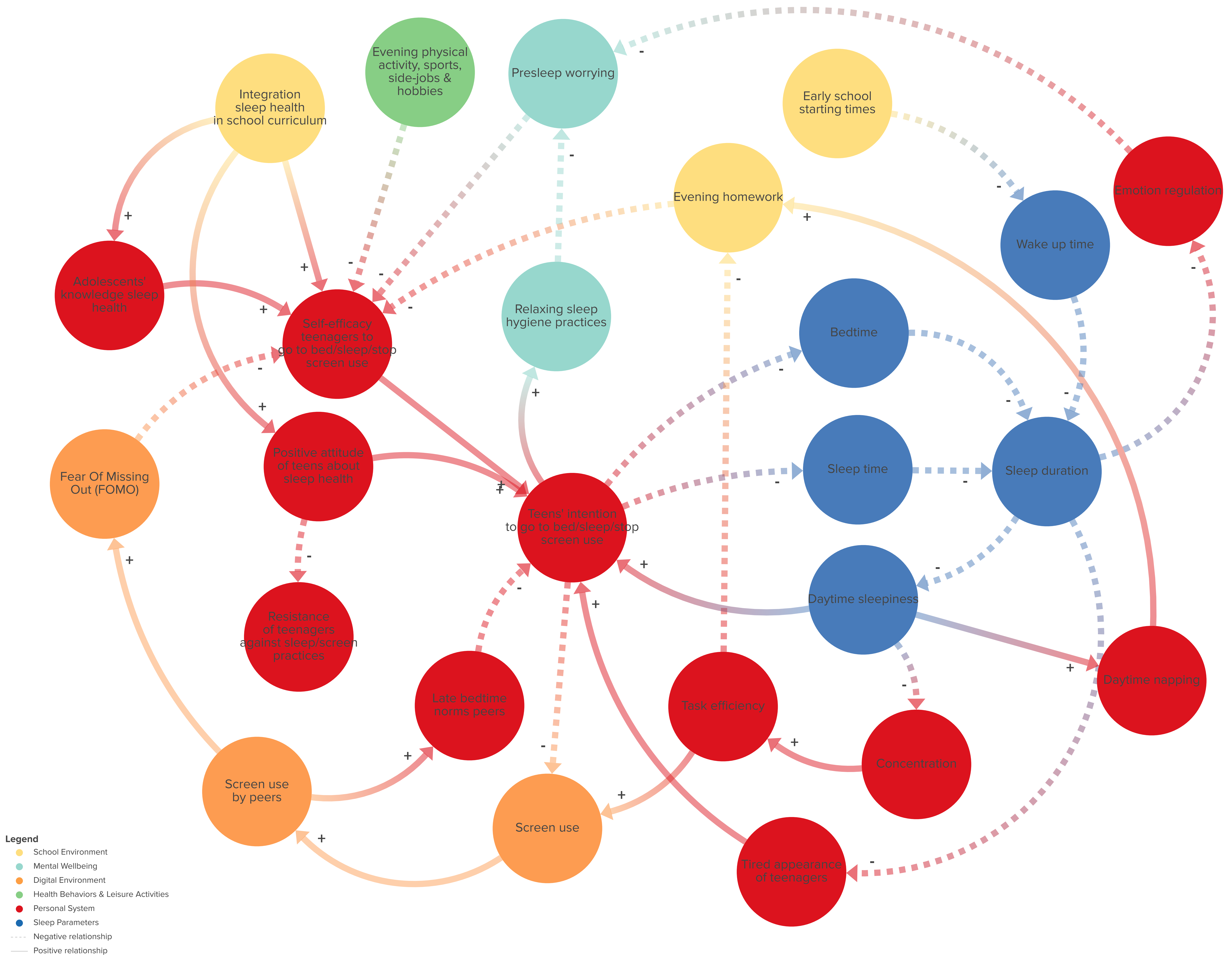
Integration sleep health in school curriculum
Evening physical activity, sports, side-jobs & hobbies
Presleep worrying
Early school starting times
Evening homework
Emotion regulation
Wake up time
Adolescents' knowledge sleep health
Self-efficacy teenagers to go to bed/sleep/stop screen use
Relaxing sleep hygiene practices
Bedtime
Fear Of Missing Out (FOMO)
Positive attitude of teens about sleep health
Teens' intention to go to bed/sleep/stop screen use
Sleep time
Sleep duration
Daytime sleepiness
Resistance of teenagers against sleep/screen practices
Late bedtime norms peers
Task efficiency
Concentration
Daytime napping
Screen use by peers
Screen use
Tired appearance of teenagers
Legend
School Environment
Mental Wellbeing
Digital Environment
Health Behaviors & Leisure Activities
Personal System
Sleep Parameters
Negative relationship
Positive relationship
